# Normal mode-guided transition pathway generation in proteins

**DOI:** 10.1371/journal.pone.0185658

**Published:** 2017-10-11

**Authors:** Byung Ho Lee, Sangjae Seo, Min Hyeok Kim, Youngjin Kim, Soojin Jo, Moon-ki Choi, Hoomin Lee, Jae Boong Choi, Moon Ki Kim

**Affiliations:** 1 School of Mechanical Engineering, Sungkyunkwan University, Suwon, Republic of Korea; 2 Department of Materials Chemistry, Nagoya University, Nagoya, Japan; 3 School of Computational Sciences, Korea Institute for Advanced Study, Seoul, Republic of Korea; 4 SKKU Advanced Institute of Nanotechnology (SAINT), Sungkyunkwan University, Suwon, Republic of Korea; University of Minnesota Twin Cities, UNITED STATES

## Abstract

The biological function of proteins is closely related to its structural motion. For instance, structurally misfolded proteins do not function properly. Although we are able to experimentally obtain structural information on proteins, it is still challenging to capture their dynamics, such as transition processes. Therefore, we need a simulation method to predict the transition pathways of a protein in order to understand and study large functional deformations. Here, we present a new simulation method called normal mode-guided elastic network interpolation (NGENI) that performs normal modes analysis iteratively to predict transition pathways of proteins. To be more specific, NGENI obtains displacement vectors that determine intermediate structures by interpolating the distance between two end-point conformations, similar to a morphing method called elastic network interpolation. However, the displacement vector is regarded as a linear combination of the normal mode vectors of each intermediate structure, in order to enhance the physical sense of the proposed pathways. As a result, we can generate more reasonable transition pathways geometrically and thermodynamically. By using not only all normal modes, but also in part using only the lowest normal modes, NGENI can still generate reasonable pathways for large deformations in proteins. This study shows that global protein transitions are dominated by collective motion, which means that a few lowest normal modes play an important role in this process. NGENI has considerable merit in terms of computational cost because it is possible to generate transition pathways by partial degrees of freedom, while conventional methods are not capable of this.

## Introduction

Proteins are essential components of living cells. Each protein has its own biological function, which is accompanied by conformational change of the protein. Therefore, studying this conformational change is necessary to understand the underlying mechanism of its biological functions. Various experimental methods have been developed as part of this effort. Specifically, NMR [1,2], Raman spectroscopy [3,4], electron cryo-microscopy [5,6], atomic force microscopy [7,8], and terahertz time-domain spectroscopy [9,10], as well as time-resolved methods such as x-ray scattering [11,12], the transient grating method [13–15] and light scattering [16] have expanded our understanding of the relationship between structure and function in biomolecules. Although the local vibrational movement of a protein in meta-stable states can be relatively easily captured by those experimental methods, it is still challenging to observe its global dynamics experimentally. This is due to the existence of a high-energy barrier between two conformational states, and also because capturing a very dynamic transition state is difficult with current experimental techniques. Thus, various computational attempts have sought to find intermediate structures from the known stable structures.

Molecular dynamics (MD) simulation [17,18] is a representative computational method that can be used to analyze the dynamics of proteins in atomic detail. However, the conventional MD simulation is inappropriate for predicting large-scale transitions due to its computational cost, despite recent efforts to overcome time limitations [19–21]. Instead, the prediction of transition pathways based on a simplified potential function, called the elastic network model (ENM), has flourished in recent years. Unlike MD simulation, which integrates an empirical potential function, ENM exploits a Hookean potential function and can significantly reduce the computational cost. The ENI first attempted to find intermediate structures by interpolating the distance between two end-point conformations of a target protein [22–24]. This transition pathway generation technique was already implemented on an online morph server called KOSMOS [25]. The mixed elastic network model (MENM) was also developed to study large-scale conformational transitions [26]. In the MENM method, the Boltzmann-weighted Go potentials for the end-point structures are combined into a smooth potential function, which interpolates conformation. The interpolated ENM (iENM) was proposed by constructing a double-well potential function from the ENMs of two end-point structures [27]. To improve conventional coarse-grained ENM-based methods without the loss of physical reality, the hybrid ENI considers the rigidity information of conformation when generating transition pathways [28]. In addition, the ANMPathway used two end-point ENMs and constructed two-state potential resulting in a cusp hypersurface [29]. The minimum energy conformation on this cusp hypersurface is regarded as the transition conformation.

Meanwhile, normal mode analysis (NMA) based on coarse-grained modeling has addressed predicting collective motions at low frequency and succeeded in explaining biological functions in terms of the collective motion [30–34]. Since the transition process mostly shows the large collective motion, there have been efforts to employ low-frequency mode shapes predicted by NMA to the transition pathway. Firstly, collective MD iteratively obtains the transition pathways by combining NMA based on ENM, which guides the collective dynamics with MD evaluating local dynamics and atomic interactions [35]. The optimized torsion-angle normal modes in internal coordinates generated more accurate transition pathways than the Cartesian modes [36]. Next, in the anisotropic network model-Monte Carlo (ANM-MC) method, normal modes are also used for describing intermediate structures on transition. Then, MC algorithm is applied to minimize their conformational energy in order to predict the transition pathway successfully [37]. Such NMA-based transition pathway prediction tools were also addressed through online web servers. NMSim reproduces the feasible transition pathways using rigid-cluster NMA in Cartesian coordinates [38], while iMODS performs the pathway generation based on NMA in internal coordinates [39].

In this work, we present a new simulation method based on ENM, which is called the normal mode guided elastic network interpolation (NGENI). This method can generate pathways on a large-scale transition process by iteratively performing NMA. The conventional ENI method generates conformational pathways between the initial and the final conformations by obtaining displacement vectors iteratively toward a direction which linearly reduces root-mean-square deviation (RMSD) between the two given structures [22–24]. On the other hand, the displacement vectors in NGENI are composed of a linear combination of normal mode vectors. Because of this methodological difference, NGENI generates theoretically more reliable pathway than ENI of which the pathway doesn’t take into account any physical aspects such as the intrinsic thermal vibration, but only complies with the given topological constraints in Cartesian space. As the existing morphing methods such as MENM, iENM and ANMPathway also utilize either topological constraints or potential energy landscape for prediction of large-scale transition pathways, our method is expected to be a new solution considering both aspects in a balanced way. In addition, NGENI is able to adjust degrees of freedom in the simulation by determining the number of normal modes to be used. This enables us to reduce the computational cost. The validity of NGENI has been verified with extensive testing by comparing NGENI to ENI.

## Materials and methods

### A set of proteins

In this work, we choose a set of 8 proteins for which two end-point structures are available at the Protein Data Bank (PDB). The RMSD between the two end-point structures was more than 3 Å for all tested proteins. Such a topological difference is enough to test whether a protein undergoes conformational changes that cause its own biological functions. The information about these proteins is listed in Table 1.

**Table 1:** Fundamental information about a set of 8 large-scale transition proteins.

Protein	PDB ID A^a^	PDB ID B^b^	No. of residues (*n*)	RMSD (Å)	No. of iterations^c^ (*s*)	Resolution^d^ (Å)
T4 lysozyme	178L	256L	162	3.4	34	1.80
Maltodextrin binding protein	1OMP	1ANF	370	3.8	38	1.67
D-allose binding protein	1GUD	1RPJ	288	4.5	45	1.70
LAO binding protein	2LAO	1LST	238	4.7	47	1.80
5’-nucleotidase	1OID	1OI8	525	5.5	55	2.10
Ribose-binding protein	1BA2	2DRI	271	6.2	62	1.60
Adenylate kinase	4AKE	1AKE	214	7.1	71	2.00
Ribonuclease III	1YZ9	1YYO	438	7.3	73	2.10

^a^ Initial conformation of each protein.

^b^ Final conformation of each protein.

^c^ The number of iterations (no. of iterations) is simply determined by multiplying the RMSD value by 10 since NGENI generates the intermediate conformations, which have an RMSD of 0.1 Å to the previous step at each iteration.

^d^ The smaller resolution value between the two conformations of each protein is selected as a representative here.

### Outline of NGENI

**Fig 1:**
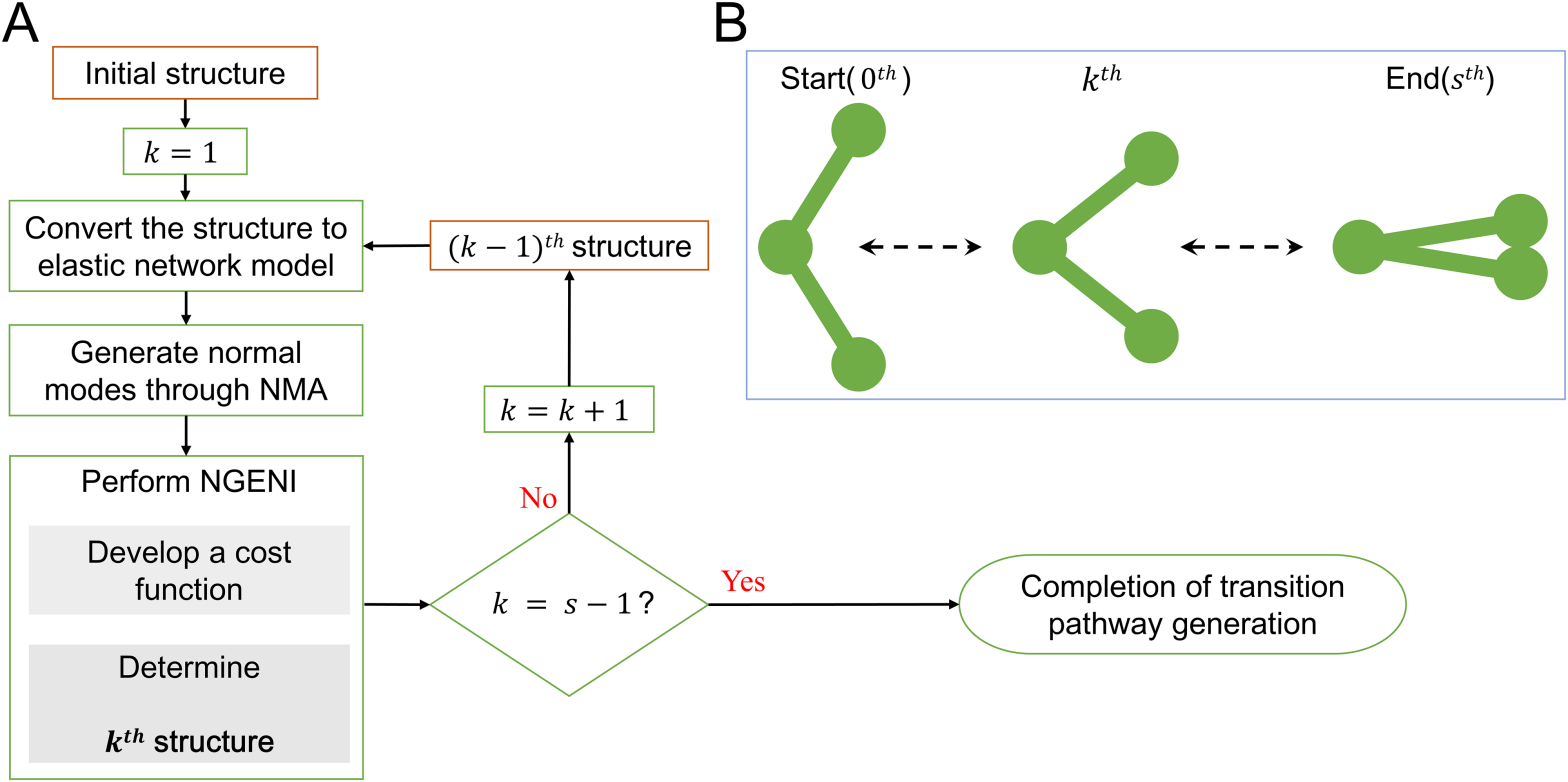
Schematic of the proposed NGENI method. (A) A flow chart of NGENI. If the total number of iterations is *s*, then *k* increases iteratively from 1 to *s*-1. (B) A schematic description of a large-scale transition pathway. Specifically, the left figure labeled ‘Start’ refers to an initial structure, the right one labeled ‘End’ refers to a final structure, and the center one labeled ‘*k*^th^’ indicates an intermediate structure at the *k*^th^ step.

The purpose of the NGENI method is to construct a pathway between two end-point structures based on low-frequency modes, which are most relevant to biological function. The pathway comprises consecutive displacement vectors between an intermediate structure and the next one. To obtain these vectors, we established an objective function in which potential energy linearly decreases with respect to the RMSD value by interpolating the distance between spatially close residues (more detailed description on RMSD calculation is given in S1 Text). Here, the coordinates are collected from the position of the alpha carbon atoms (C_α_s), and spatially close residues are connected by linear springs based on the ENM concept [40,41]. The key idea of the NGENI is to generate transition pathways based on normal mode vectors. A linear combination of normal mode vectors yields the corresponding intermediate structure. By adjusting the contribution weighting of each mode vector, we can define the vector set that satisfies the desired value of potential energy. Iteratively, a series of these displacement vectors are obtained from the initial to the final conformation to create a possible transition pathway. Here, the number of iterations is preset by multiplying the RMSD value between the two end-point structures by 10 in order to get a smooth transition pathway with the RMSD increment of 0.1 Å every iteration step. The overall scheme of NGENI is shown in Fig 1 and more details about the proposed objective function are also described in the following chapter.

### Cost function

For a protein composed of *n* residues (e.g., C_α_s based on coarse-grained ENM), the coordinates for the two end-point structures are denoted by {*x*_*i*_} and {*y*_*i*_}, respectively. When we use the *m* lowest normal modes in the simulation, we can define the displacement vector for the *i*^th^ residue as follows

$$\delta_{i}=c_{1}v_{i,1}+c_{2}v_{i,2}+\cdots +c_{m}v_{i,m}=V_{i}C_{w}\in R^{3},$$ (1)

where *v*_*i*,*m*_ is the *m*^th^ normal mode vector of the *i*^th^ residue and *c*_*m*_ is a weighting constant of the *m*^th^ normal mode. Thus, we can define

$$V_{i}=\left[v_{i,1},v_{i,2},\cdots ,v_{i,m}\right]\in R^{3\times m}$$ (2)

and

$$C_{w}={\left[c_{1},c_{2},\cdots ,c_{m}\right]}^{T}\in R^{m}.$$ (3)

Here, we introduce a cost function as follows

$$\text{C}\left(C_{w}\right)=\frac{1}{2}\sum_{i=1}^{n-1}\sum_{j=i+1}^{n}k_{i,j}{\left\{\left\|x_{i}+V_{i}C_{w}-x_{j}-V_{j}C_{w}\right\|-l_{i,j}\right\}}^{2},$$ (4)

where *k*_*i*,*j*_ is an element of the linking matrix which contains binary information about virtual spring connection in ENM. The value of *l*_*i*,*j*_ is the desired distance between the *i*^th^ residue and *j*^th^ residue at a certain intermediate state. The value of *l*_*i*,*j*_ can be determined as

$$l_{i,j}=\left(1-\alpha \right)\left\|x_{i}-x_{j}\right\|+\alpha \left\|y_{i}-y_{j}\right\|,$$ (5)

where α is a scale factor that interpolates between the initial distance ‖*x*_*i*_ − *x*_*j*_‖ and the final one ‖*y*_*i*_ − *y*_*j*_‖. It ranges from 0 (i.e., initial) to 1 (i.e., final) with an increment of 1/*s*. Again, *s* is the total number of iterations here.

We simplify the cost function in Eq (4) in order to obtain the optimum solution of *C*_*w*_. First, we define *C*_*i*,*j*_ as a part of the cost function

$$C_{i,j}=\frac{1}{2}k_{i,j}{\left\{\left\|x_{i}+V_{i}C_{w}-x_{j}-V_{j}C_{w}\right\|-l_{i,j}\right\}}^{2}.$$ (6)

This equation can be simplified into a quadratic equation in terms of *C*_*w*_ by using a Taylor series approximation for small values of *V*_*i*_*C*_*w*_ and V_*j*_*C*_*w*_.

$$\left\|x+VC_{w}\right\|\approx \left\|x\right\|+\frac{x\cdot VC_{w}}{\left\|x\right\|}+\frac{1}{2}\frac{{\left(VC_{w}\right)}^{T}A\left(x\right)VC_{w}}{\left\|x\right\|},$$ (7)

where

$$\text{A}\left(x\right)=E_{3}-\frac{xx^{T}}{{\left\|x\right\|}^{2}},$$ (8)

and *E*_3_ is the 3 by 3 identity matrix.

Then, we write Eq (6) as

$$C_{i,j}=\frac{1}{2}k_{i,j}\left(C_{i,j}^{\left(1\right)}+C_{i,j}^{\left(2\right)}+C_{i,j}^{\left(3\right)}\right),$$ (9)

where

$$C_{i,j}^{\left(1\right)}={\left(V_{i}C_{w}-V_{j}C_{w}\right)}^{T}\left[E_{3}-l_{i,j}\frac{A\left(x_{i}-x_{j}\right)}{\left\|x_{i}-x_{j}\right\|}\right]\left(V_{i}C_{w}-V_{j}C_{w}\right),$$ (10)

$$C_{i,j}^{\left(2\right)}=2\left(1-\frac{l_{i,j}}{\left\|x_{i}-x_{j}\right\|}\right){\left(x_{i}-x_{j}\right)}^{T}\left(V_{i}C_{w}-V_{j}C_{w}\right),$$ (11)

and

$$C_{i,j}^{\left(3\right)}={\left(x_{i}-x_{j}\right)}^{T}\left(x_{i}-x_{j}\right)-2l_{i,j}\left\|x_{i}-x_{j}\right\|+l_{i,j}^{2}.$$ (12)

In $C_{i,j}^{\left(1\right)}$ in Eq (10), we define $P_{i,j}^{\left(1\right)}\in R^{3\times 3}$ as

$$P_{i,j}^{\left(1\right)}=\left[E_{3}-l_{i,j}\frac{A\left(x_{i}-x_{j}\right)}{\left\|x_{i}-x_{j}\right\|}\right].$$ (13)

Then, we can rewrite

$$C_{i,j}^{\left(1\right)}={\left(V_{i}C_{w}-V_{j}C_{w}\right)}^{T}P_{i,j}^{\left(1\right)}\left(V_{i}C_{w}-V_{j}C_{w}\right).$$ (14)

Considering all the spring connections, we can obtain the following equation

$$\frac{1}{2}\sum_{i=1}^{n-1}\sum_{j=i+1}^{n}k_{i,j}C_{i,j}^{\left(1\right)}=\frac{1}{2}C_{w}{}^{T}\Lambda^{\left(1\right)}C_{w},$$ (15)

where Λ^(1)^ ∈ *R*^*m*×*m*^ is defined as

$$\Lambda^{\left(1\right)}=\sum_{i=1}^{n-1}\sum_{j=i+1}^{n}k_{i,j}\left(V_{i}^{T}P_{i,j}^{\left(1\right)}V_{i}-V_{i}^{T}P_{i,j}^{\left(1\right)}V_{j}-V_{j}^{T}P_{i,j}^{\left(1\right)}V_{i}+V_{j}^{T}P_{i,j}^{\left(1\right)}V_{j}\right).$$ (16)

Next, for $C_{i,j}^{\left(2\right)}$ in Eq (11), taking $P_{i,j}^{\left(2\right)}\in R^{1\times 3}$ as

$$P_{i,j}^{\left(2\right)}=2\left(1-\frac{l_{i,j}}{\left\|x_{i}-x_{j}\right\|}\right){\left(x_{i}-x_{j}\right)}^{T}.$$ (17)

Then,

$$C_{i,j}^{\left(2\right)}=P_{i,j}^{\left(2\right)}\left(V_{i}C_{w}-V_{j}C_{w}\right).$$ (18)

We can also simplify the term such that

$$\frac{1}{2}\sum_{i=1}^{n-1}\sum_{j=i+1}^{n}k_{i,j}C_{i,j}^{\left(2\right)}=\frac{1}{2}\Lambda^{\left(2\right)}C_{w},$$ (19)

where Λ^(2)^ ∈ *R*^1×*m*^ is

$$\Lambda^{\left(2\right)}=\sum_{i=1}^{n-1}\sum_{j=i+1}^{n}k_{i,j}P_{i,j}^{\left(2\right)}\left(V_{i}-V_{j}\right).$$ (20)

Lastly, let Λ^(3)^ be a constant such that

$$\Lambda^{\left(3\right)}=\sum_{i=1}^{n-1}\sum_{j=i+1}^{n}k_{i,j}C_{i,j}^{\left(3\right)}.$$ (21)

Consequently, we can derive a quadratic form of the cost function by substitution of Eqs (16), (20), and (21) into (9).

$$\text{C}\left(C_{w}\right)=\sum_{i=1}^{n-1}\sum_{j=i+1}^{n}C_{i,j}\approx \frac{1}{2}C_{w}{}^{T}\Lambda^{\left(1\right)}C_{w}+\frac{1}{2}\Lambda^{\left(2\right)}C_{w}+\Lambda^{\left(3\right)}.$$ (22)

Our goal is to determine the value of *C*_*w*_, which minimizes Eq (22). To do that, the *C*_*w*_ has to satisfy the following equation

$$\frac{\partial C\left(C_{w}\right)}{\partial C_{w}}=\Lambda^{\left(1\right)}C_{w}+\frac{1}{2}{\left(\Lambda^{\left(2\right)}\right)}^{T}=0.$$ (23)

Through this computation, we can obtain the optimum weighting constant *C*_*w*_ and determine the optimum displacement vectors from an intermediate state to the next one. Iteratively, this process can generate a conformational transition pathway between the given two end-point structures. From a computational point of view, the whole process can be divided into two parts: NMA and the main computational part from Eqs (9) to (23). First, the time complexity of NMA is O(*nm*^2^) when using the “eigs” function in MATLAB. This function is well designed to find the largest/smallest magnitude eigenvalues of sparse matrix efficiently using Krylov subspace methods including Lanczos and Arnoldi algorithms [42,43]. Next, in the main computational part, the most computational effort is required to construct the cost function in Eq (16) with the time complexity of O(*nm*^2^). Consequently, the overall time complexity of optimum-NGENI can be expressed as O(*n*) when *m* is a constant. On the other hand, in ENI, the computation time is mainly consumed by multiplication/inversion of large and sparse matrix with the time complexity of O(*n*^2^) (see S2 Text for further details).

## Results and discussion

### Verification of NGENI by using the full degrees of freedom

We first evaluated the performance of NGENI with all normal modes (full-NGENI) by comparing it with the conventional ENI pathways [22–24] in terms of average RMSD values. For 8 large-scale transition proteins, we obtained average RMSD which is the average of RMSD values between two corresponding intermediate conformations generated by full-NGENI and conventional ENI for all iteration steps. As a result, the negligibly small RMSD values indicate that the full-NGENI generated similar pathways to those of conventional ENI for all cases (Table 2). This is because the full normal mode vectors can take the complete set of degrees of freedom (DOF) into account. Mathematically speaking, this is nothing more than a different representation of the bases that constitute the given topological space. Therefore, we confirmed that the full-NGENI could generate reliable pathways for a large-scale transition process based on the fact that the conventional ENI pathway has already been verified elsewhere [22–24].

**Table 2:** Comparison between the full-NGENI and the conventional ENI pathways.

Protein	Resolution (Å)	Average RMSD^a^ (Å)
T4 lysozyme	1.80	0.0879
Maltodextrin binding protein	1.67	0.0296
D-allose binding protein	1.70	0.0012
LAO binding protein	1.80	0.0002
5’-nucleotidase	2.10	0.0021
Ribose-binding protein	1.60	0.0031
Adenylate kinase	2.00	0.0029
Ribonuclease III	2.10	0.1620

^a^ It indicates the averaged RMSD value between the full-NGENI and the conventional ENI pathways. Here, all the ENI pathways are automatically generated through KOSMOS online server [25].

### Potential of NGENI with partial degrees of freedom

**Fig 2:**
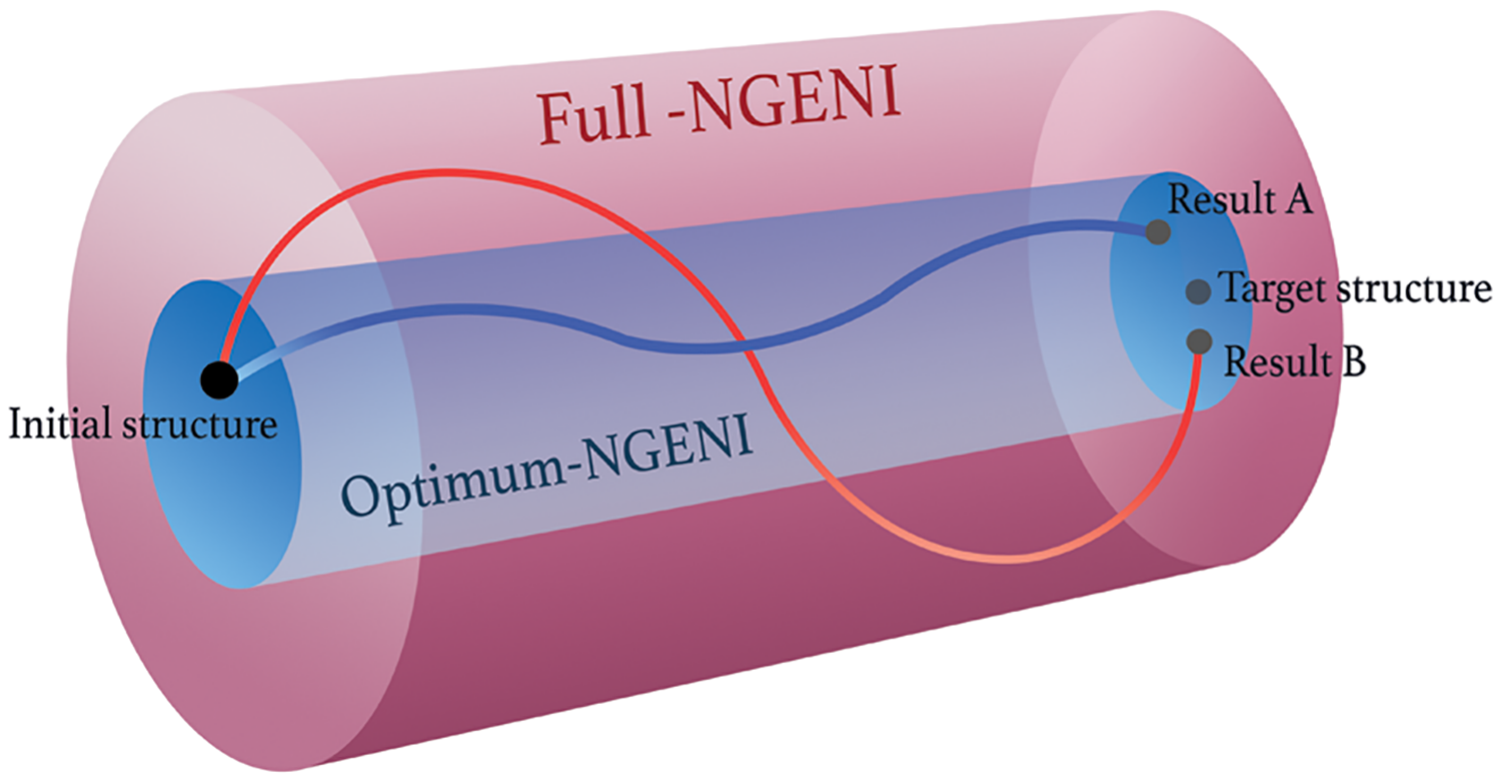
A schematic of different NGENI pathways in terms of degrees of freedom. A cylindrical tube represents the searching space for a transition pathway depicted by a curved line. The optimum-NGENI is shown in blue, while the full-NGENI is shown in red. Both pathways are generated from the same initial and target structures.

As low frequency modes dominantly show collective motion, one can significantly reduce computational cost without loss of generality of the pathway by using only those modes. Here, we define another NGENI with partial DOF, called optimum-NGENI, and test whether the optimum-NGENI is still able to generate reasonable transition pathways. Fig 2 illustrates this concept, in which each curved line represents a transition pathway inside a cylindrical space spanned by corresponding normal modes. The smaller search space of the optimum-NGENI enables us to dramatically reduce the computational cost. The size of the searching space can be easily adjusted by the number of normal modes taken in the optimum-NGENI, but it has not been determined whether this optimum number satisfies all general cases.

To determine the optimum number of lowest normal modes used in the optimum-NGENI, the quality of the optimum-NGENI pathway was evaluated using RMSD between intermediate conformations and the final given structure for every iteration step. If these RMSD values are less than its experimental resolution, then the proposed optimum-NGENI with a particular number of lowest normal modes is considered to satisfy the convergence condition. Although the optimal number of normal modes may be different in each case, 30 lowest normal modes seem to be sufficient to generate reliable pathways, as shown in Table 3.

**Table 3:** Convergence of the optimum-NGENI.

Protein	Resolution (Å)	Convergence condition, m^a^					
		5	10	20	30	40	Full
T4 lysozyme	1.80	O	O	O	O	O	O
Maltodextrin binding protein	1.67	X	O	O	O	O	O
D-allose binding protein	1.70	O	O	O	O	O	O
LAO binding protein	1.80	O	O	O	O	O	O
5’-Nucleotidase	2.10	X	X	X	O	O	O
Ribose-binding protein	1.60	O	O	O	O	O	O
Adenylate Kinase	2.00	O	O	O	O	O	O
Ribonuclease III	2.10	X	O	O	O	O	O

If the convergence condition is satisfied, it is marked ‘O’. If not, it is marked ‘X’.

^a^ The number of lowest normal modes used in the optimum-NGENI test. The full-NGENI case is also listed in the last column as a reference.

**Fig 3:**
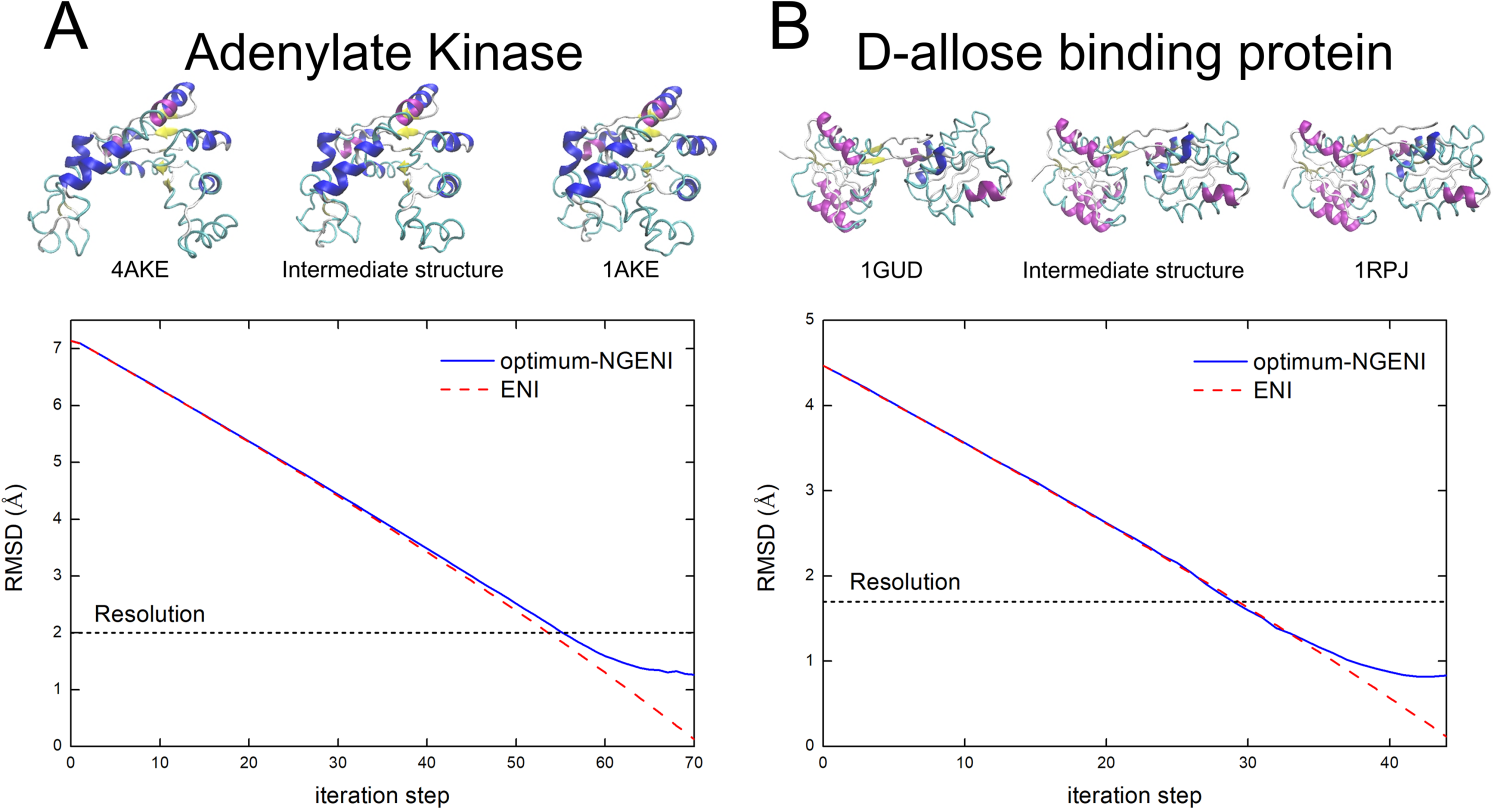
RMSD comparison between the optimum-NGENI and the conventional ENI pathways for adenylate kinase and D-allose binding protein. (A) Adenylate kinase, (B) D-allose binding protein. The upper figures show rough transition pathways of proteins using representative intermediate structures. The lower graphs show changes in RMSD between the final structure and intermediate conformations generated by two different methods: full-NGENI (dashed red) and optimum-NGENI (solid blue). The black dotted line represents experimental resolution of each protein.

For further assessment of this convergence condition, Fig 3 presents the transition pathways of two proteins: adenylate kinase and D-allose binding protein. Adenylate kinase catalyzes the transfer of a phosphoryl group from ATP to AMP [44] and undergoes rigid body motions of the NMP_bind_ and LID domains with two pairs of hinges connecting each domain to CORE domain [45]. Fig 3A (upper) includes an actual simulation result showing that the optimum-NGENI successfully generates the rigid body movements of adenylate kinase. In addition, both full-NGENI and optimum-NGENI pathways are compared to each other in Fig 3A (lower). Unlike full-NGENI, the error of the optimum-NGENI pathway is accumulated at the end and obviously caused by the missing DOF. However, this error is acceptable compared to the experimental resolution of the adenylate kinase structure. This result suggests that only a small portion of the lowest normal modes is sufficient to predict transition pathways without loss of generality because this portion has enough information to comprehend biologically important collective protein motion. Fig 3B also confirms similar results for D-allose binding protein, which has three hinge points between two domains [46]. The upper figure in Fig 3B shows that the generated pathway demonstrates the hinge movement without difficulty, and the lower one verifies that the pathway of optimum-NGENI meets the convergence condition. Furthermore, their reverse transition pathways (from closed to open form) were also generated by optimum-NGENI. They not only satisfy the convergence condition but also preserve realistic geometry during the reverse transition (see S3 Text).

### Availability of optimum-NGENI for large proteins

Thus far, we have defined optimum-NGENI as the NGENI using only the first 30 lowest normal modes through the evaluation of convergence condition of generated transition pathways. Now, we test if optimum-NGENI is still able to generate transition pathways for relatively large proteins such as group II chaperonin. The detailed structural information of group II chaperonin is provided in Table 4.

**Table 4:** Structural information of group II chaperonin.

Protein	PDB ID A	PDB ID B	No. of residues (*n*)	RMSD (Å)	No. of iterations (*s*)	Resolution (Å)
Group II chaperonin	3IYF	3J03	3928	15.4	154	4.80

**Fig 4:**
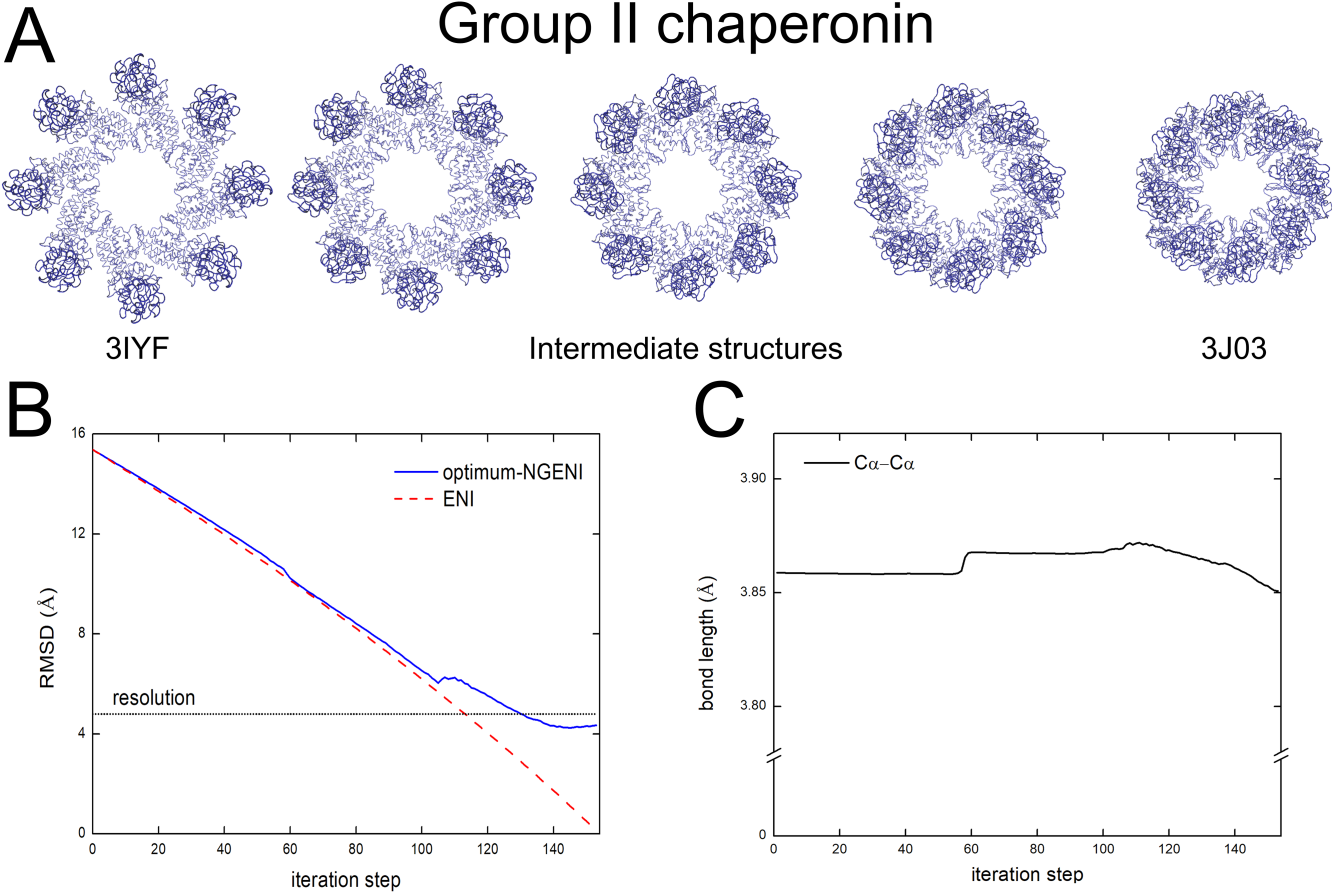
The optimum-NGENI pathway of group II chaperonin. (A) Transition pathway from 3IYF (open) to 3J03 (closed). (B) RMSD comparison between optimum-NGENI (solid blue) and ENI (dashed red). The black dotted line represents experimental resolution. (C) Variation of bond length (solid black) during the conformational change.

As shown in Fig 4A, the optimum-NGENI pathway successfully describes the hinge-bending motions of the intermediate domains which play a key role in the folding mechanism of the group II chaperonin. Moreover, RMSD values of optimum-NGENI and ENI are compared to each other in Fig 4B. Although the optimum-NGENI pathway shows higher RMSD error at the end stage, it can still converge below the experimental resolution. Lastly, the bond lengths and bond angles are measured for evaluating physical reality of the proposed pathway (Fig 4C). We have also confirmed that difference with the two end-point structures is negligible for bond length (less than 0.03Å).

Ideally speaking, there is no size limitation to optimum-NGENI. For large system, we can expect much higher computational efficiency by using a finite number of meaningful normal modes as the driving force of pathway generation. Although there is still an argument on how to predetermine the number of normal modes required to capture the system dynamics without any loss of generality, empirically speaking (also supported by our case study results), the first 30 lowest normal modes are enough to generate the transition pathway successfully. Also, it is noted that this number is not determined by the size of protein structure but the complexity of conformational transition.

### Quality of optimum-NGENI pathway

**Fig 5:**
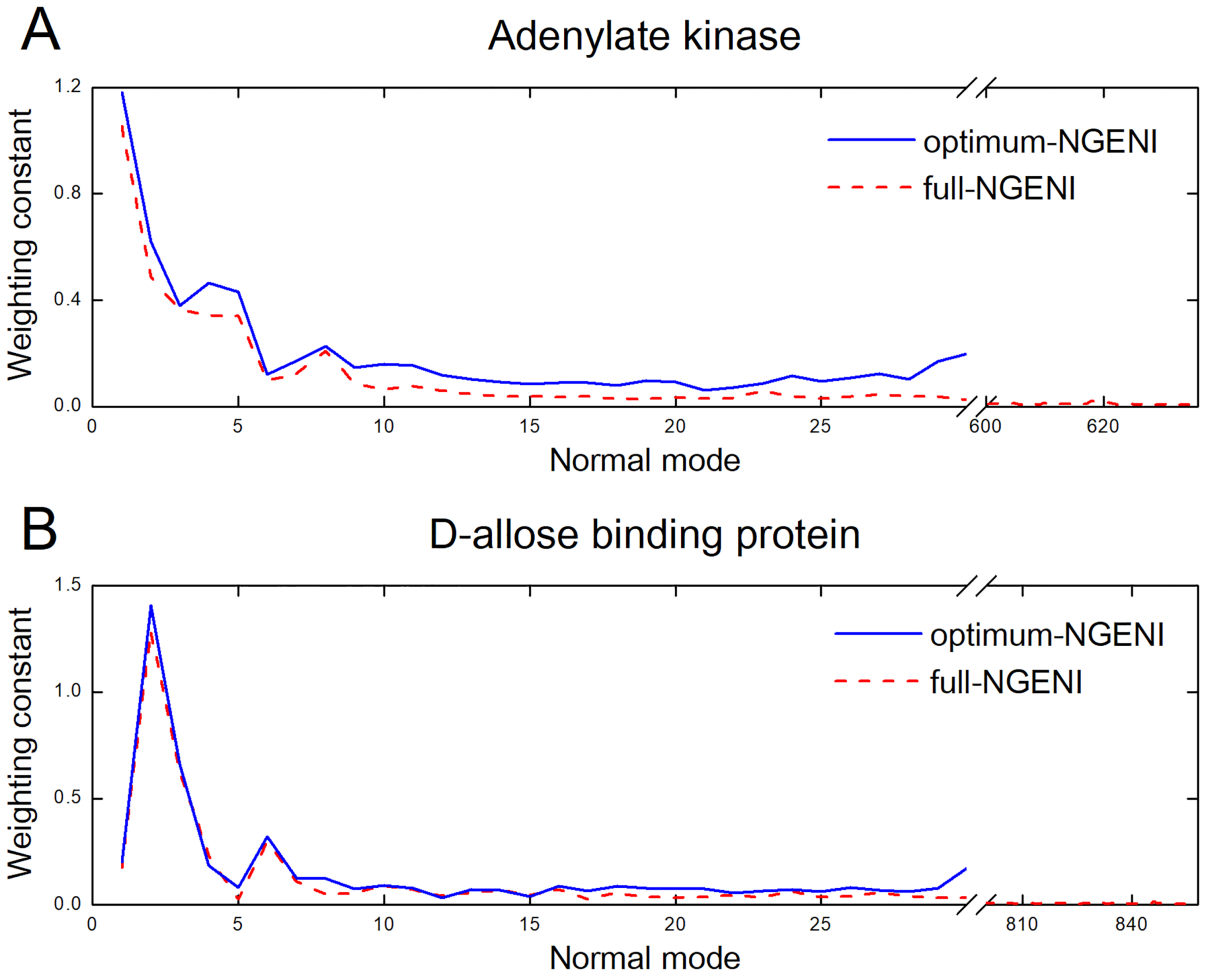
Comparison of weighting constants between the full-NGENI and the optimum-NGENI. (A) Adenylate kinase, (B) D-allose binding protein. Weighting constants of normal modes for the full-NGENI (red dashed line) and the optimum-NGENI (blue solid line) are compared to each other. They represent the average values of weighting constants for all iteration steps (optimum-NGENI: modes 1 to 30, full-NGENI: all modes).

To address whether the optimum-NGENI method achieves goals as good as the conventional ENI, the performance of both methods is compared using the average RMSD values for our protein set including the group II chaperonin (Table 5). Here, these values are obtained from averaging RMSD values between two corresponding intermediate conformations generated by optimum-NGENI and ENI for every iteration step. As the average value is smaller than the corresponding resolution value for all cases, topological difference between two pathways is negligible. To evaluate quality of the optimum-NGENI pathways for adenylate kinase and D-allose binding protein, we compared their weighting constants with those of full-NGENI (Fig 5). For both cases, the first 30 weighting constants of the full-NGENI were very similar to those of the optimum-NGENI, in the sense that several lowest modes dominantly influence the transition pathway in proteins by generating large-scale and collective motion. Quantitatively speaking, the correlation coefficients between the two methods are 0.988 and 0.992 for adenylate kinase and D-allose binding protein, respectively. This result indeed validates that the proposed optimum-NGENI method can generate transition pathways as good as the conventional ENI does with only the fixed number of lowest normal modes (i.e., 30 in this context). The weighting constants for all the other protein pathways are also provided in S1 Fig.

**Table 5:** Comparison between the optimum-NGENI and the conventional ENI pathways.

Protein	Resolution (Å)	Average RMSD^a^ (Å)
T4 lysozyme	1.80	0.30
Maltodextrin binding protein	1.67	0.58
D-allose binding protein	1.70	0.40
LAO binding protein	1.80	0.36
5’-nucleotidase	2.10	1.20
Ribose-binding protein	1.60	0.34
Adenylate kinase	2.00	0.55
Ribonuclease III	2.10	0.42
Group II chaperonin	4.80	2.09

^a^ The average RMSD indicates the average of RMSD values between intermediate conformations generated by optimum-NGENI and conventional ENI for all iteration steps.

### Computational complexity of optimum-NGENI

The main advantage of NGENI is that one can incorporate large collective motions effectively when predicting transition pathways in proteins, because NGENI generates transition pathways considering geometric constraints and physical mechanics, despite the simple interpolation method. Of course, the computational cost of NGENI is higher than that of ENI because NGENI has to perform NMA at every iteration step to update intermediate conformations. Using a finite number of normal modes, however, the optimum-NGENI can overcome this drawback because it drastically reduces computational burden in the main computation in which the next intermediate conformation is determined by displacement vectors obtained from NMA. A rough mathematical calculation with big O notation yields that the optimum-NGENI follows O(*nm*^*2*^), whereas the conventional ENI follows O(*n*^*2*^) where *n* is the number of residues and *m* is a constant number of normal modes utilized in optimum-NGENI (see Materials and methods and S2 Text for more details). As the size of protein increases, the conventional ENI method requires much more computational time than optimum-NGENI.

**Fig 6:**
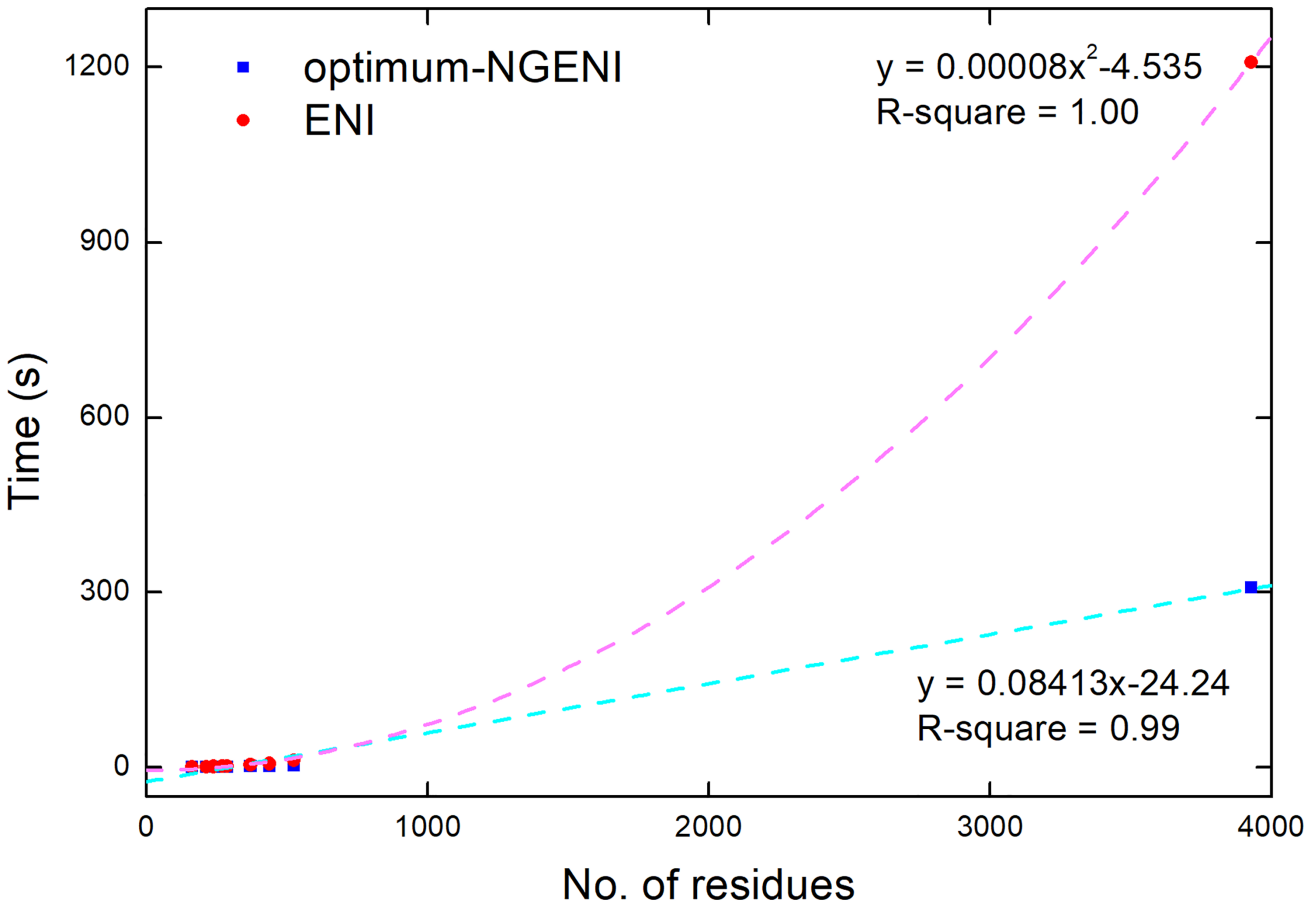
Computational cost comparison between optimum-NGENI and ENI. For 9 example proteins, the average computation time for each iteration step is measured and individually marked with respect to protein size. Optimum-NGENI (the conventional ENI) is denoted by blue quadrangles (red circles) and the curve fitting lines are also added for both methods.

This relationship is verified by comparison of the actual computation time for both methods. For appropriate comparison, we take into account the average computing time to obtain each intermediate conformation. Fig 6 shows that computation time of the ENI (denoted by red circles) grows quadratically with respect to protein size, while the corresponding computation time of optimum-NGENI (denoted by blue quadrangles) increases linearly. Therefore, the optimum-NGENI method can be a reliable alternative to the conventional ENI by balancing physical realism and computational cost, regardless of protein size.

## Conclusions

There are significant challenges in using experimental techniques to capture temporally lengthy, large-scale protein dynamics at the atomic level, so simulation methods play an important role in filling this gap by generating transition pathways between different conformational states, which are strongly related to biological functions. However, there is still concern regarding simulation reliability and computational cost. To compensate for this weakness, this work proposes a new morphing method called NGENI that interpolates the distance between spatially close residues based on a linear combination of normal mode vectors. This key idea helps us generate topologically allowable and physically reliable pathways.

Furthermore, the optimum-NGENI successfully provides in-depth study on transition pathway generation. First, it can elucidate how well a minimum number of collective modes generate protein transition pathways. Second, the concept of the optimum weighting constant can be also interpreted as a quantitative measure of the contribution of each mode to the transition pathway. Third, it compromises computational cost with the physical realism of the generated transition pathway by taking only a fixed number of lowest normal modes as a basis for searching space.

Consequently, it is expected that the optimum-NGENI not only assesses degrees of collectivity in protein dynamics, but also captures its functional transition pathway through a linear combination of several intrinsic vibration modes.

## Supporting information

S1 Text Details on the root-mean-square deviation (RMSD). (DOCX)

S2 Text Details on the computational cost of the NGENI described by big O notation. (DOCX)

S3 Text A review of reverse transition pathways from closed to open form generated by optimum-NGENI. (DOCX)

S1 Fig Weighting constants of optimum-NGENI. (A) T4 lysozyme, (B) Maltodextrin binding protein, (C) D-allose binding protein, (D) LAO binding protein, (E) 5’-nucleotidase, (F) Ribose-binding protein, (G) Adenylate kinase, (H) Ribonuclease III, (I) Group II chaperonin. The values described in the graphs represent average weighting constants for all iteration steps.
(TIF)

S2 Fig Density map of linking matrix. (A) D-allose binding protein (B) Group II chaperonin.
(TIF)

S3 Fig RMSD comparison between original and reverse pathways for adenylate kinase and D-allose binding protein. (A,B,C) Adenylate kinase, (D,E,F) D-allose binding protein. The graphs show changes in RMSD between the target structure and an intermediate conformation for the two pathways: original pathway (dashed red) and reverse pathway (solid blue). Three different methods are used to generate transition pathways: (A,D) optimum-NGENI, (B,E) 100-NGENI using the 100 lowest normal modes, and (C,F) full-NGENI. The black dotted line represents experimental resolution of each protein.
(TIF)

S1 Table Density of linking matrix for the set of proteins. (DOCX)

S2 Table Variation of bond length over the reverse pathways of adenylate kinase and D-allose binding protein. (DOCX)

## References

[1] Mittermaier A, Kay LE. Review—New tools provide new insights in NMR studies of protein dynamics. Science. 2006;312: 224–228. 16614210 10.1126/science.1124964

[2] Boehr DD, Dyson HJ, Wright PE. An NMR perspective on enzyme dynamics. Chem Rev. 2006;106: 3055–3079. doi: 10.1021/cr050312q 16895318 10.1021/cr050312q

[3] He L, Rodda T, Haynes CL, Deschaines T, Strother T, Diez-Gonzalez F, et al Detection of a foreign protein in milk using surface-enhanced Raman spectroscopy coupled with antibody-modified silver dendrites. Anal Chem. 2011;83: 1510–1513. doi: 10.1021/ac1032353 21306123 10.1021/ac1032353

[4] Brown KG, Erfurth SC, Small EW, Peticolas WL. Conformationally dependent low-frequency motions of proteins by laser Raman spectroscopy. Proc Natl Acad Sci USA. 1972;69: 1467–1469. 4504361 10.1073/pnas.69.6.1467 PMC426727

[5] Chiu W, Baker ML, Jiang W, Dougherty M, Schmid MF. Electron cryomicroscopy of biological machines at subnanometer resolution. Structure. 2005;13: 363–372. doi: 10.1016/j.str.2004.12.016 15766537 10.1016/j.str.2004.12.016

[6] Henderson R, Baldwin JM, Ceska TA, Zemlin F, Beckmann E, Downing KH. Model for the structure of bacteriorhodopsin based on high-resolution electron cryo-microscopy. J Mol Biol. 1990;213: 899–929. doi: 10.1016/S0022-2836(05)80271-2 2359127 10.1016/S0022-2836(05)80271-2

[7] Pfreundschuh M, Alsteens D, Hilbert M, Steinmetz MO, Müller DJ. Localizing chemical groups while imaging single native proteins by high-resolution atomic force microscopy. Nano Lett. 2014;14: 2957–2964. doi: 10.1021/nl5012905 24766578 10.1021/nl5012905

[8] Fotiadis D. Imaging and manipulation of biological structures with the AFM. Micron. 2002;33: 385–397. 11814877 10.1016/s0968-4328(01)00026-9

[9] Acbas GK, Niessen A, Snell EH, Markelz AG. Optical measurements of long-range protein vibrations. Nat Commun. 2014;5: 3076 doi: 10.1038/ncomms4076 24430203 10.1038/ncomms4076

[10] Turton DA, Senn HM, Harwood T, Lapthorn AJ, Ellis EM, Wynne K. Terahertz underdamped vibrational motion governs protein-ligand binding in solution. Nat Commun. 2014;5: 3999 doi: 10.1038/ncomms4999 24893252 10.1038/ncomms4999

[11] Shi Y. A glimpse of structural biology through X-ray crystallography. Cell. 2014;159: 995–1014. doi: 10.1016/j.cell.2014.10.051 25416941 10.1016/j.cell.2014.10.051

[12] Blanchet CE, Svergun DI. Small-angle X-Ray scattering on biological macromolecules and nanocomposites in solution. Annu Rev Phys Chem. 2013;64: 37–54. doi: 10.1146/annurev-physchem-040412-110132 23216378 10.1146/annurev-physchem-040412-110132

[13] Eitoku T, Zarate X, Kozhukh GV, Kim JI, Song PS, Terazima M. Time-resolved detection of conformational changes in oat phytochrome A: Time-dependent diffusion. Biophys J. 2006;91: 3797–3804. doi: 10.1529/biophysj.106.092882 16935954 10.1529/biophysj.106.092882 PMC1630454

[14] Nada T, Terazima M. A novel method for study of protein folding kinetics by monitoring diffusion coefficient in time domain. Biophys J. 2003;85: 1876–1881. doi: 10.1016/S0006-3495(03)74615-3 12944300 10.1016/S0006-3495(03)74615-3 PMC1303359

[15] Terazima M, Hirota N. Translational diffusion of a transient radical studied by the transient grating method, pyrazinyl radical in 2-propanol. J Chem Phys. 1993;98: 6257 doi: 10.1063/1.464819

[16] Sarkar HK, Moon DK, Song PS, Chang T, Yu H. Tertiary structure of phytochrome probed by quasi-elastic light scattering and rotational relaxation time measurements. Biochemistry. 1984;23: 1882–1888. doi: 10.1021/bi00303a046

[17] Levitt M, Hirshberg M, Sharon R, Daggett V. Potential energy function and parameters for simulations of the molecular dynamics of proteins and nucleic acids in solution. Comp Phys Commun. 1995;91: 215–231. doi: 10.1016/0010-4655(95)00049-L

[18] Levitt M. Molecular dynamics of native protein. J Mol Biol. 1983;168: 595–617. doi: 10.1016/S0022-2836(83)80304-0 6193280 10.1016/s0022-2836(83)80304-0

[19] Leckband D. Design rules for biomolecular adhesion: Lessons from force measurements. Annu Rev Chem Biomol. 2010;1: 365–389. doi: 10.1146/annurev-chembioeng-073009-100931 22432586 10.1146/annurev-chembioeng-073009-100931

[20] Adcock SA, McCammon JA. Molecular dynamics: Survey of methods for simulating the activity of proteins. Chem Rev. 2006;106: 1589–1615. doi: 10.1021/cr040426m 16683746 10.1021/cr040426m PMC2547409

[21] Elber R. Long-timescale simulation methods. Curr Opin Struc Biol. 2005;15: 151–156. doi: 10.1016/j.sbi.2005.02.004 15837172 10.1016/j.sbi.2005.02.004

[22] Kim MK, Chirikjian GS, Jernigan RL. Elastic models of conformational transitions in macromolecules. J Mol Graph Model. 2002;21: 151–160. doi: 10.1016/S1093-3263(02)00143-2 12398345 10.1016/s1093-3263(02)00143-2

[23] Kim MK, Jernigan RL, Chirikjian GS. Efficient generation of feasible pathways for protein conformational transitions. Biophys J. 2002;83: 1620–1630. doi: 10.1016/S0006-3495(02)73931-3 12202386 10.1016/S0006-3495(02)73931-3 PMC1302259

[24] Kim MK, Jernigan RL, Chirikjian GS. Rigid-cluster models of conformational transitions in macromolecular machines and assemblies. Biophys J. 2005;89: 43–55. doi: 10.1529/biophysj.104.044347 15833998 10.1529/biophysj.104.044347 PMC1366543

[25] Seo S, Kim MK. KOSMOS: a universal morph server for nucleic acids, proteins and their complexes. Nucleic Acids Res. 2012;40: W531–W536. doi: 10.1093/nar/gks525 22669912 10.1093/nar/gks525 PMC3394317

[26] Zheng W, Brooks BR, Hummer G. Protein conformational transitions explored by mixed elastic network models. Proteins. 2007;69: 43–57. doi: 10.1002/prot.21465 17596847 10.1002/prot.21465

[27] Tekpinar M, Zheng W. Predicting order of conformational changes during protein conformational transitions using an interpolated elastic network model. Proteins. 2010;78: 2469–2481. doi: 10.1002/prot.22755 20602461 10.1002/prot.22755

[28] Seo S, Jang Y, Qian P, Liu WK, Choi JB, Lim BS, et al Efficient prediction of protein conformational pathways based on the hybrid elastic network model. J Mol Graph Model. 2014;47: 25–36. doi: 10.1016/j.jmgm.2013.10.009 24296313 10.1016/j.jmgm.2013.10.009

[29] Das A, Gur M, Cheng MH, Jo S, Bahar I, Roux B. Exploring the conformational transitions of biomolecular systems using a simple two-state anisotropic network model. PLoS Comput Biol. 2014;10: e1003521 doi: 10.1371/journal.pcbi.1003521 24699246 10.1371/journal.pcbi.1003521 PMC3974643

[30] Bahar I, Lezon TR, Yang LW, Eyal E. Global dynamics of proteins: bridging between structure and function. Annu Rev Biophys. 2010;39: 23–42. doi: 10.1146/annurev.biophys.093008.131258 20192781 10.1146/annurev.biophys.093008.131258 PMC2938190

[31] Yang L, Song G, Jernigan RL. How well can we understand large-scale protein motions using normal modes of elastic network models? Biophys J. 2007;93: 920–929. doi: 10.1529/biophysj.106.095927 17483178 10.1529/biophysj.106.095927 PMC1913142

[32] Bahar I, Rader AJ. Coarse-grained normal mode analysis in structural biology. Curr Opin Struc Biol. 2005;15: 586–592. doi: 10.1016/j.sbi.2005.08.007 16143512 10.1016/j.sbi.2005.08.007 PMC1482533

[33] Tama F, Sanejouand YH. Conformational change of proteins arising from normal mode calculations. Protein Eng Des Sel. 2001;14: 1–6. doi: 10.1093/protein/14.1.1 11287673 10.1093/protein/14.1.1

[34] Kim MH, Lee BH, Kim MK. Robust elastic network model: A general modeling for precise understanding of protein dynamics. J Struct Biol. 2015;190: 338–347. doi: 10.1016/j.jsb.2015.04.007 25891099 10.1016/j.jsb.2015.04.007

[35] Gur M, Madura JD, Bahar I. Global transitions of proteins explored by a multiscale hybrid methodology: Application to adenylate kinase. Biophys J. 2013;105: 1643–1652. doi: 10.1016/j.bpj.2013.07.058 24094405 10.1016/j.bpj.2013.07.058 PMC3791301

[36] Bray JK, Weiss DR, Levitt M. Optimized torsion-angle normal modes reproduce conformational changes more accurately than cartesian modes. Biophys J. 2011;101: 2966–2969. doi: 10.1016/j.bpj.2011.10.054 22208195 10.1016/j.bpj.2011.10.054 PMC3244061

[37] Uyar A, Kantarci-Carsibasi N, Haliloglu T, Doruker P. Features of large hinge-bending conformational transitions. Prediction of closed structrue from open state. Biophys J. 2014;106: 2656–2666. doi: 10.1016/j.bpj.2014.05.017 24940783 10.1016/j.bpj.2014.05.017 PMC4070069

[38] Krüger DM, Ahmed A, Gohlke H. NMSim Web Server: integrated approach for normal mode-based geometric simulations of biologically relevant conformational transitions in proteins. Nucleic Acids Res. 2012;40: W310–W316. doi: 10.1093/nar/gks478 22669906 10.1093/nar/gks478 PMC3394247

[39] López-Blanco JR, Aliga JI, Quintana-Ortí ES, Chacón P. iMODS: internal coordinates normal mode analysis server. Nucleic Acids Res. 2014;42: W271–W276. doi: 10.1093/nar/gku339 24771341 10.1093/nar/gku339 PMC4086069

[40] Atilgan AR, Durell SR, Jernigan RL, Demirel MC, Keskin O, Bahar I. Anisotropy of fluctuation dynamics of proteins with an elastic network model. Biophys J. 2001;80: 505–515. doi: 10.1016/S0006-3495(01)76033-X 11159421 10.1016/S0006-3495(01)76033-X PMC1301252

[41] Bahar I, Atilgan AR, Erman B. Direct evaluation of thermal fluctuations in proteins using a single-parameter harmonic potential. Fold Des. 1997;2: 173–181. doi: 10.1016/S1359-0278(97)00024-2 9218955 10.1016/S1359-0278(97)00024-2

[42] Athanasios CA, Danny CS, Serkan G. A survey of model reduction methods for large linear systems. Contemp Math. 2001;280: 193–216.

[43] Lee J, Balakrishnan V, Koh CK, Jiao D. From O(k2N) to O(N): A Fast Complex-Valued Eigenvalue Solver For Large-Scale On-Chip Interconnect Analysis. IEEE T Microw Theory. 2009;12: 3219–3228.

[44] Maragakis P, Karplus M. Large amplitude conformational change in proteins explored with a plastic network model: Adenylate kinase. J Mol Biol. 2005;352: 807–822. doi: 10.1016/j.jmb.2005.07.031 16139299 10.1016/j.jmb.2005.07.031

[45] Müller CW, Schlauderer GJ, Reinstein J, Schulz GE. Adenylate kinase motions during catalysis: an energetic counterweight balancing substrate binding. Structure. 1996;4: 147–156. 8805521 10.1016/s0969-2126(96)00018-4

[46] Magnusson U, Chaudhuri BN, Ko J, Park C, Jones TA, Mowbray SL. Hinge-bending motion of D-allose-binding protein from Escherichia coli: three open conformations. J Biol Chem. 2002;277: 14077–14084. doi: 10.1074/jbc.M200514200 11825912 10.1074/jbc.M200514200

