# Supplementary material for: Normal mode-guided transition pathway generation in proteins
Source: PLoS One. 2017 Oct 11;12(10):e0185658. doi: 10.1371/journal.pone.0185658 (PMC5636086; doi:10.1371/journal.pone.0185658)
Supplement: S1 Text — (DOCX) [file pone.0185658.s001.docx]

**S1 Text**

**Root-mean-square deviation (RMSD)**

RMSD is a typical metric describing the spatial difference between two comparable structures as the average distance between two corresponding residues in protein. In the NGENI simulation, the structural similarity or difference between each intermediate conformation and the final structure is evaluated using this RMSD calculation.

For this calculation, two given structures must be properly placed by RMS superposition. We can understand RMS superposition is a process to define the translation vector, $\vec{d}$, and rotation matrix, $R$, to minimize the RMS error such that

$$E^{2}\left( R,\vec{d} \right)=\sum_{i}^{n} \left\| \vec{x}_{i}-\left( R\vec{Y}_{i}+\vec{d} \right) \right\|^{2}, (1)$$

where {$\vec{x}_{i}$} and {$\vec{Y}_{i}$} are the set of coordinates consisting of *n* residues (where $i=1, \cdots,$*n*). First, one can translate each structure into its center of mass position so that two structures can share the origin resulting that $\vec{d}$ becomes a trivial zero vector after translation. Next, to obtain the rotation matrix, $R$, we first construct $\hat{R}$ [1] such that

$$\hat{R}=\left[ \sum_{s=0}^{n} \vec{x}_{s}{\vec{Y}_{s}}^{T} \right]\left[ \sum_{k=0}^{n} \vec{Y}_{k}{\vec{Y}_{k}}^{T} \right]^{-1}. (2)$$

Then, $R$ can be obtained using $\hat{R}$ in the following form

$$R=\hat{R}\left( \hat{R}^{T}\hat{R} \right)^{-0.5}. (3)$$

Once we get the new optimum structural information $\{\vec{y}_{i}\}$ such that

$$\vec{y}_{i}=R\vec{Y}_{i}+\vec{d}, (4)$$

RMSD of these two conformations ($x_{i}$ and $y_{i}$) can be obtained by the following simple calculation

$$RMSD=\sqrt{\frac{1}{n}\sum_{j=1}^{n} \left\| x_{j}-y_{j} \right\|^{2}}. (5)$$

**References**

1. Kim MK, Chirikjian GS, Jernigan RL. Elastic models of conformational transitions in macromolecules. J Mol Graph Model. 2002;21: 151–160. doi: 10.1016/S1093-3263(02)00143-2. PMID:12398345
