# Supplementary material for: Normal mode-guided transition pathway generation in proteins
Source: PLoS One. 2017 Oct 11;12(10):e0185658. doi: 10.1371/journal.pone.0185658 (PMC5636086; doi:10.1371/journal.pone.0185658)
Supplement: S2 Text — (DOCX) [file pone.0185658.s002.docx]

**S2 Text**

**Computational cost of NGENI algorithm described by big O notation**

Big O notation is a mathematical calculation used in computer science to describe the computational cost or complexity of algorithms. Big O defines computational burden of the algorithms by describing their asymptotic behaviors. For example, a typical matrix multiplication between an *p* by *q* matrix and an *q* by *r* matrix requires O(*pqr*).

In the NGENI algorithm, the computation process from Eq (10) to Eq (23) can be regarded as the main computation to obtain displacement vectors based on the proposed cost function. Among them, Eq (16) requires the most computational complexity.

$$\Lambda^{\left( 1 \right)}=\sum_{i=1}^{n-1} \sum_{j=i+1}^{n} k_{i,j}\left( V_{i}^{T}P_{i,j}^{\left( 1 \right)}V_{i}-V_{i}^{T}P_{i,j}^{\left( 1 \right)}V_{j}-V_{j}^{T}P_{i,j}^{\left( 1 \right)}V_{i}+V_{j}^{T}P_{i,j}^{\left( 1 \right)}V_{j} \right) (16)$$

Suppose that the optimum-NGENI method using *m* normal modes is applied to a protein with *n* residues. In Eq (16), any matrix multiplication in form of $V_{i}^{T}P_{i,j}^{\left( 1 \right)}V_{j}$ follows O(*m^2^*). Here, $V_{i}$ is a 3 by *m* matrix and $P_{i,j}^{\left( 1 \right)}$ is a 3 by 3 matrix. Moreover, this calculation must be repeated *n*^2^ times by the double summation, so that the total computational cost of Eq (16) is O(*n*^2^*m*^2^) (i.e., O(*n*^2^) because *m* is fixed to 30 in optimum-NGENI). In contrast, the conventional ENI requires O(*n*^3^) in the course of matrix multiplication and inversion to obtain the displacement vectors.

However, the actual computation yields totally different behaviors. Both optimum-NGENI and ENI follow time complexity of O(*n*) and O(*n*^2^), respectively, through the least squares regression line fitting of actual computation time data for 9 protein structures including group II chaperonin (Fig 6). To analyze the cause of the difference between the actual time complexity and its theoretical value, we focused on the density of linking matrix, $k$, which contains binary information about connectivity of ENM. As shown in S1 Table, the density of linking matrix is proportional to *n*^-1^ because a residue in space can create elastic network only with surrounding neighbors. To further illustrate, linking matrices of the two proteins (D-allose binding protein and group II chaperonin) are also provided in S2 Fig. This figure also apparently shows that spring connection in group II chaperonin is much sparser than that of D-allose binding protein in spite of its overwhelming number of spring connections. Consequently, the density of linking matrix should be taken into account together when calculating time complexity because it degrades the order of theoretical value so that we can get O(*n*) and O(*n*^2^) for optimum-NGENI and ENI, respectively, in the actual measurement of computation time.
