# Supplementary material for: Normal mode-guided transition pathway generation in proteins
Source: PLoS One. 2017 Oct 11;12(10):e0185658. doi: 10.1371/journal.pone.0185658 (PMC5636086; doi:10.1371/journal.pone.0185658)
Supplement: S3 Text — (DOCX) [file pone.0185658.s003.docx]

**S3 Text**

**Analysis of reverse transition pathways generated by optimum-NGENI**

In general, transition pathways are generated from open to closed structure unless otherwise noticed. However, to test the ability of optimum-NGENI, reverse transition is considered here for the next two proteins: adenylate kinase and D-allose binding protein. The quality of reverse transition pathway from closed to open structure is also evaluated in terms of the convergence condition and physical reality. We first ran three different NGENI simulations such as optimum-NGENI, 100-NGENI using the 100 lowest normal modes, and full-NGENI to test the convergence condition. As shown in S3 Fig, it is not surprising that the more normal modes are used, the more both original and reverse pathways are stable. Although the reverse pathways don’t look as good as the original ones, they are still acceptable even in case of optimum-NGENI. The instability of the reverse pathways seems to be ordinary because the initial structures are in closed form. Fundamentally, compared to open form, it is rarely observed that a single hinge mode dominates the entire mode shapes of closed form because its elastic network around the hinge domain is formed more tightly than that of open form so that more normal modes are required to reproduce hinge motion of the reverse pathway. Furthermore, we also calculated Cα−Cα bond length over the reverse pathways. As listed in S2 Table, there is no significant change which implies that structural reality is also preserved well during the reverse transition.
