# Supplementary figures and images for: Normal mode-guided transition pathway generation in proteins
Source: PLoS One. 2017 Oct 11;12(10):e0185658. doi: 10.1371/journal.pone.0185658 (PMC5636086; doi:10.1371/journal.pone.0185658)

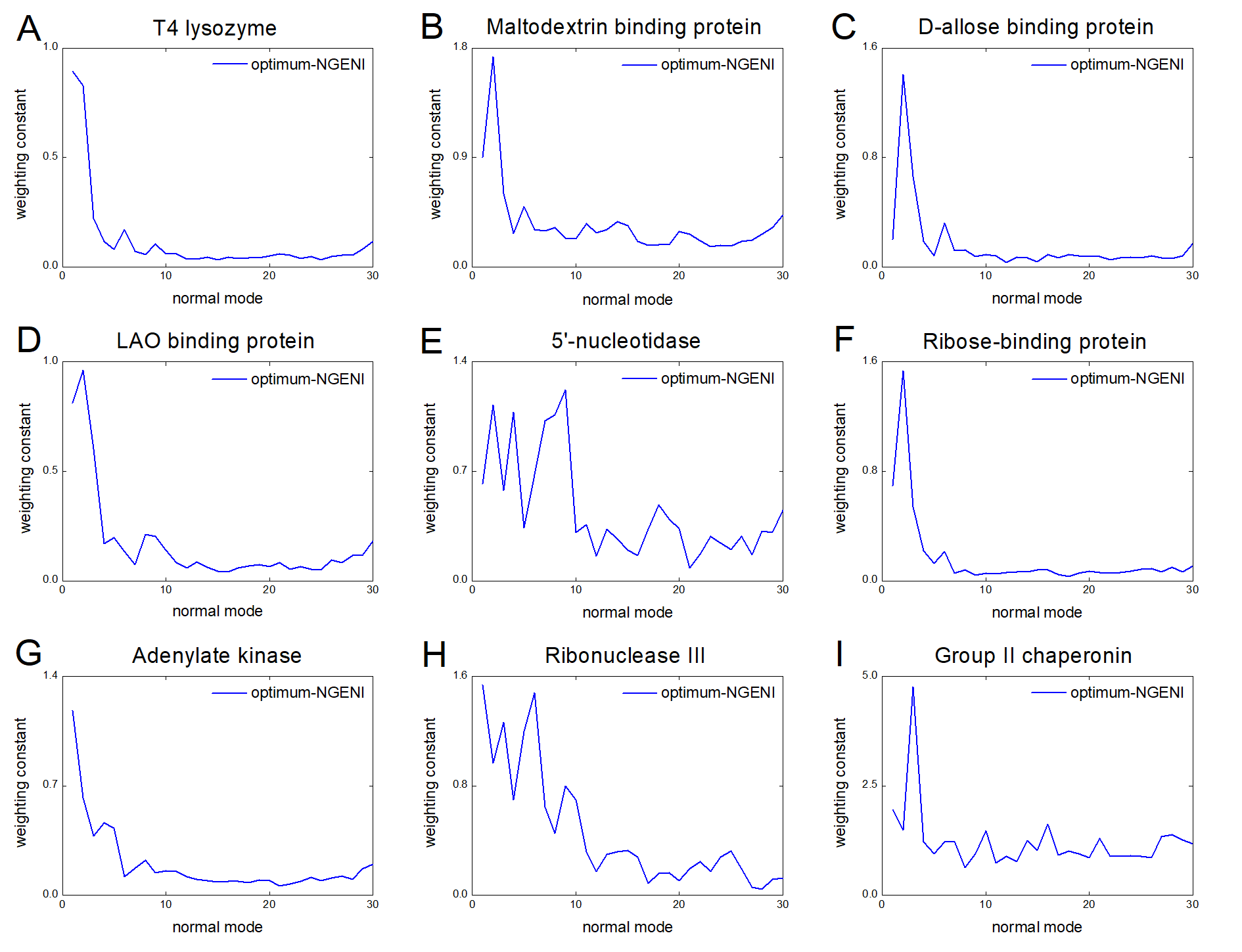

Supplement: S1 Fig — (A) T4 lysozyme, (B) Maltodextrin binding protein, (C) D-allose binding protein, (D) LAO binding protein, (E) 5’-nucleotidase, (F) Ribose-binding protein, (G) Adenylate kinase, (H) Ribonuclease III, (I) Group II chaperonin. The values described in the graphs represent average weighting constants for all iteration steps. (TIF) [file pone.0185658.s004.tif]

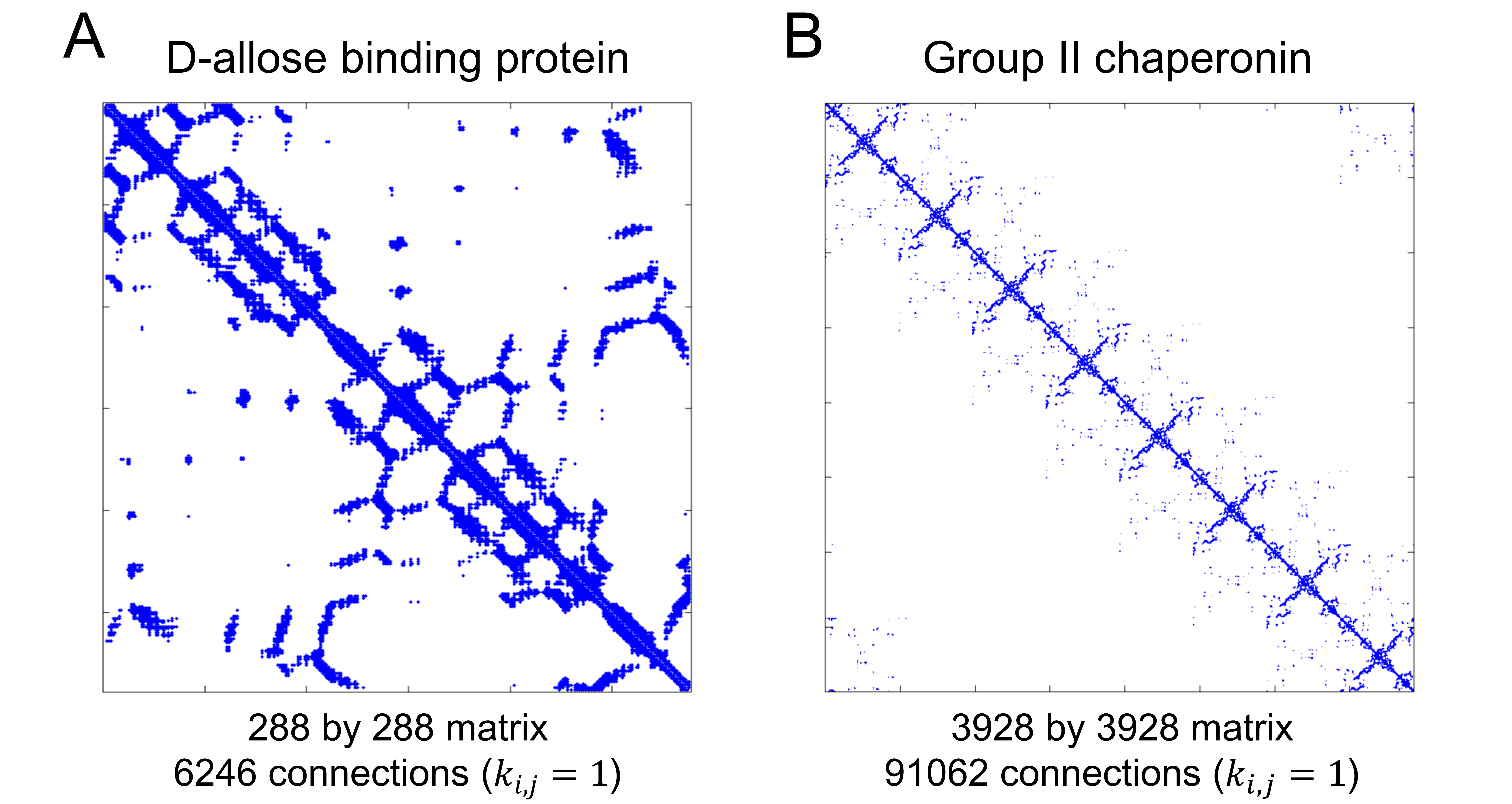

Supplement: S2 Fig — (A) D-allose binding protein (B) Group II chaperonin. (TIF) [file pone.0185658.s005.tif]

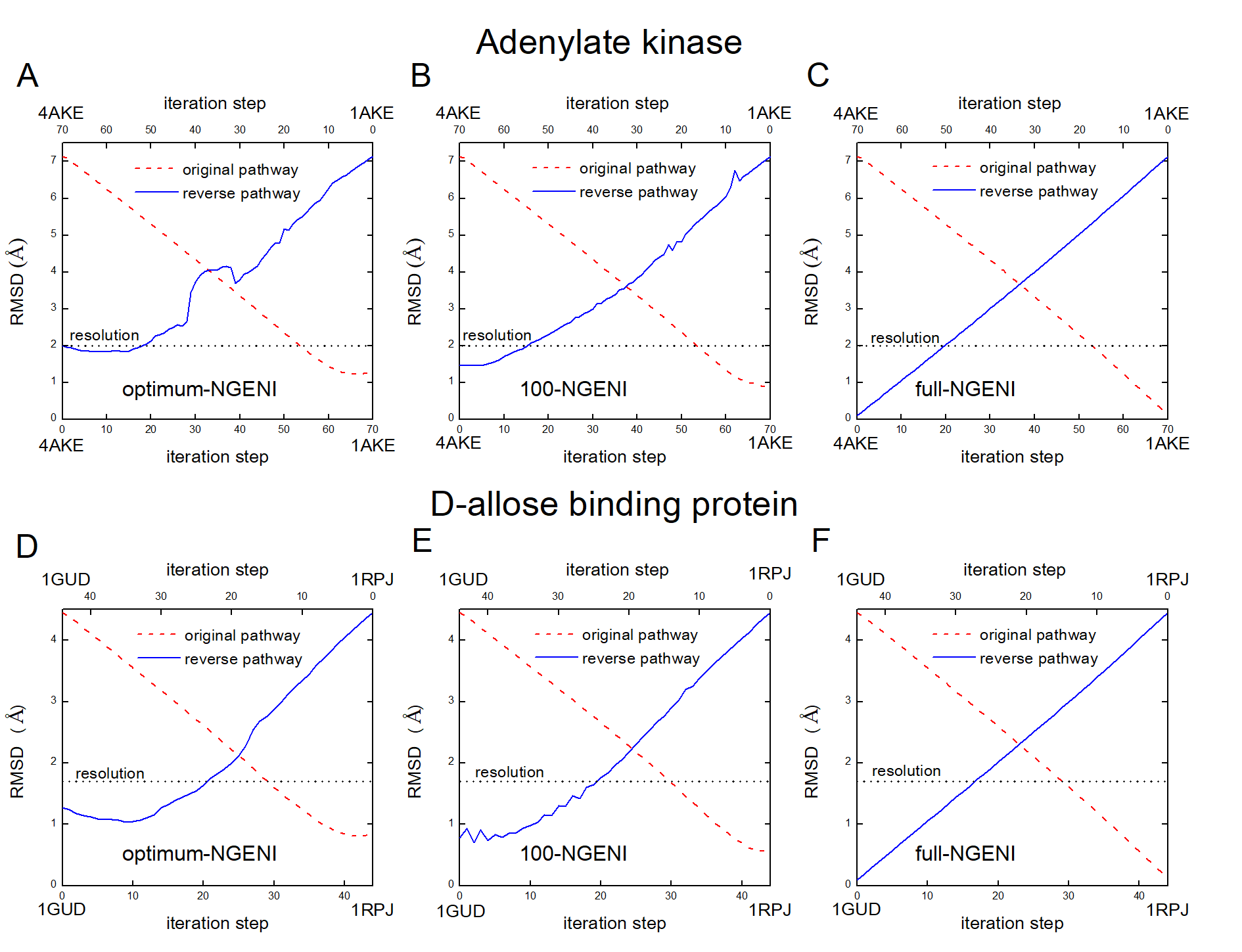

Supplement: S3 Fig — (A,B,C) Adenylate kinase, (D,E,F) D-allose binding protein. The graphs show changes in RMSD between the target structure and an intermediate conformation for the two pathways: original pathway (dashed red) and reverse pathway (solid blue). Three different methods are used to generate transition pathways: (A,D) optimum-NGENI, (B,E) 100-NGENI using the 100 lowest normal modes, and (C,F) full-NGENI. The black dotted line represents experimental resolution of each protein. (TIF) [file pone.0185658.s006.tif]
