# Supplementary material for: Normal mode-guided transition pathway generation in proteins
Source: PLoS One. 2017 Oct 11;12(10):e0185658. doi: 10.1371/journal.pone.0185658 (PMC5636086; doi:10.1371/journal.pone.0185658)
Supplement: S1 Table — (DOCX) [file pone.0185658.s007.docx]

**S1 Table. Quantitative data on virtual spring connections for the set of proteins**

| Protein | No. of residues (*n*) | No. of spring connections | Density of linking matrix^a^ |
| --- | --- | --- | --- |
| T4 lysozyme | 162 | 3530 | 0.13 |
| Maltodextrin binding protein | 370 | 7988 | 0.06 |
| D-allose binding protein | 288 | 6246 | 0.08 |
| LAO binding protein | 238 | 5282 | 0.09 |
| 5’-nucleotidase | 525 | 11220 | 0.04 |
| Ribose-binding protein | 271 | 5912 | 0.08 |
| Adenylate kinase | 214 | 4932 | 0.11 |
| Ribonuclease III | 438 | 9428 | 0.05 |
| Group II chaperonin | 3928 | 91062 | 0.01 |

**^a^ The density of linking matrix is defined as the number of spring connections divided by *n*^2^.**
