# Supplementary material for: Normal mode-guided transition pathway generation in proteins
Source: PLoS One. 2017 Oct 11;12(10):e0185658. doi: 10.1371/journal.pone.0185658 (PMC5636086; doi:10.1371/journal.pone.0185658)
Supplement: S2 Table — (DOCX) [file pone.0185658.s008.docx]

**S2 Table. Variation of bond length over the reverse pathways of adenylate kinase and D-allose binding protein**

| Bond length | 1AKE (initial) | | 4AKE (final) | | Transition pathway | |
| --- | --- | --- | --- | --- | --- | --- |
|  | Mean | Std | Mean | Std | Mean | Std |
| C_α_-C_α_ length (Å) | 3.82 | 0.07 | 3.80 | 0.06 | 4.13 | 0.20 |
| Bond length | 1RPJ (initial) | | 1GUD (final) | | Transition pathway | |
|  | Mean | Std | Mean | Std | Mean | Std |
| C_α_-C_α_ length (Å) | 3.81 | 0.02 | 3.81 | 0.02 | 3.87 | 0.04 |
